# Bis[1-(4-chloro­benz­yl)pyridinium] bis­(1,2,5-thia­diazole-3,4-dithiol­ato)nickelate(II)

**DOI:** 10.1107/S160053681102873X

**Published:** 2011-07-23

**Authors:** Zhi-min Wang, Fang-quan Wang, Zi-yang Liu

**Affiliations:** a Department of Chemistry, Zhejiang University, Hangzhou, 310027, People’s Republic of China; b College of Biology and Environment Engineering, Zhejiang Shuren University, Hangzhou 310015, People’s Republic of China

## Abstract

The asymmetric unit of the salt, (C_12_H_11_ClN)_2_[Ni(C_2_N_2_S_3_)_2_], comprises one cation and a half of Ni(tdas)_2_ (tdas = 1,2,5-thia­diazole-3,4-dithiol­ate) anion. The Ni^II^ atom is located at a centre of inversion. The Ni^II^ atom has a square-planar coordination with Ni—S distances of 2.2052 (4) and 2.1970 (5) Å. In crystal, weak C—H⋯S and C—H⋯Ni contacts are observed between the anions and cations.

## Related literature

For background to complexes containing the [Ni(mnt)_2_] anion, see: Robertson & Cronin (2002 ▶); Xie *et al.* (2002 ▶); Ni *et al.* (2005 ▶); Chen *et al.* (2010 ▶). For details of other square-planar [Ni(tdas)_2_] complexes, see: Awaga *et al.* (1994 ▶); Yamochi *et al.* (2001 ▶); Okuno *et al.* (2003 ▶); Ni *et al.* (2004 ▶). For C—H⋯Ni contacts, see: Brookhart *et al.* (2007 ▶); Yang & Ni (2006 ▶).
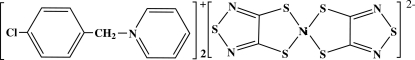


## Experimental

### 

#### Crystal data

(C_12_H_11_ClN)_2_[Ni(C_2_N_2_S_3_)_2_]
*M* *_r_* = 764.49
Monoclinic, 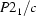

*a* = 11.3091 (10) Å
*b* = 12.6699 (12) Å
*c* = 12.2405 (11) Å
β = 116.005 (1)°
*V* = 1576.3 (2) Å^3^
*Z* = 2
Mo *K*α radiation
μ = 1.21 mm^−1^
*T* = 296 K
0.22 × 0.17 × 0.11 mm

#### Data collection

Bruker SMART APEX CCD diffractometer
Absorption correction: multi-scan (*SADABS*; Sheldrick, 2004 ▶) *T*_min_ = 0.788, *T*_max_ = 0.872
11011 measured reflections
2771 independent reflections
2583 reflections with *I* > 2σ(*I*)
*R*_int_ = 0.023

#### Refinement

*R*[*F* ^2^ > 2σ(*F* ^2^)] = 0.024
*wR*(*F* ^2^) = 0.062
*S* = 1.03
2771 reflections
196 parameters
H-atom parameters constrained
Δρ_max_ = 0.34 e Å^−3^
Δρ_min_ = −0.34 e Å^−3^

### 

Data collection: *SMART* (Bruker, 2000 ▶); cell refinement: *SAINT* (Bruker, 2000 ▶); data reduction: *SAINT*; program(s) used to solve structure: *SHELXTL* (Sheldrick, 2008 ▶); program(s) used to refine structure: *SHELXTL*; molecular graphics: *SHELXTL*; software used to prepare material for publication: *SHELXTL*.

## Supplementary Material

Crystal structure: contains datablock(s) I, global. DOI: 10.1107/S160053681102873X/kp2343sup1.cif

Structure factors: contains datablock(s) I. DOI: 10.1107/S160053681102873X/kp2343Isup2.hkl

Additional supplementary materials:  crystallographic information; 3D view; checkCIF report

## Figures and Tables

**Table 1:** Hydrogen-bond geometry (Å, °)

*D*—H⋯*A*	*D*—H	H⋯*A*	*D*⋯*A*	*D*—H⋯*A*
C4—H4⋯S2^i^	0.93	2.86	3.765 (2)	163
C11—H11⋯S2^ii^	0.93	2.80	3.715 (2)	169
C9—H9*B*⋯Ni1^iii^	0.97	2.90	3.818 (3)	159

Symmetry codes: (i) 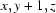; (ii) 
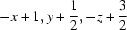
; (iii) 
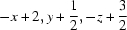
.

**Fig. 1.**
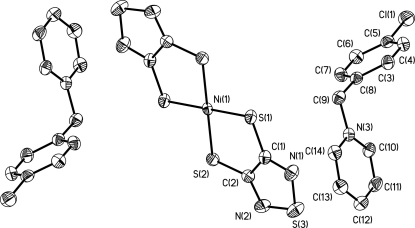
The molecular structure of (I) with atom labels of an asymmetric units and 30% probability displacement ellipsoids for non-H atoms. The complete is generated by the inversion symmetry operation: -x + 2, y + 1/2, -z + 2.

**Fig. 2.**
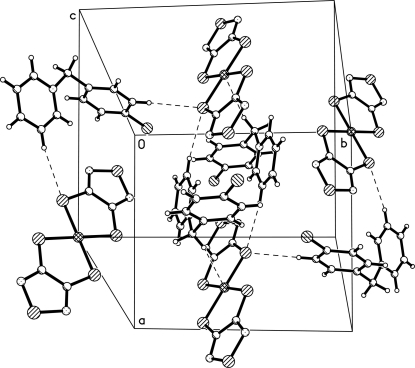
Crystal packing of (I) showing the C—H···S and C—H···Ni contacts between the anions and cations drawn as dashed lines.

## supplementary crystallographic information

### Comment

Much effort has been devoted to the study of bis(1,2-ditholene) transition metal complexes because of their extensive applications in molecular materials showing magnetic, superconducting, and optical properties. Among these complexes, [*M*(tdas)_2_]^n-^ (tdas is 1,2,5-thiadiazole-3,4-dithiolate, n is 1 or 2) complexes show interesting magnetic properties with unusual phase transition and electro-conductive properties in the solid state. As a continuation of our work in this field, we have obtained a new ion-pair complex, [4ClBzPy]_2_[Ni(tdas)_2_] (I), by introducing 1-(4'-chlorobenzyl)pyridinium as a counterion into the system containing the [Ni(tdas)_2_]^2-^ anion.

There are one [4ClBzPy]^+^ and a half of [Ni(tdas)_2_]^2-^ anion in an asymmetric unit of (I). The nickel(II) ion of Ni(tdas)_2_ anion is situated at a center of symmetry of a square planar complex (Fig. 1). The two [4ClBzPy]^+^ ions are related to each other by the symmetry centre. The Ni1—S1 and Ni1—S2 bond distances are 2.205 (1)Å and 2.197 (1) Å (Table 1), and the S1—Ni1—S2 bond angle within the five-membered ring is 93.42 (2)°, as that have been found for other [Ni(tdas)_2_]^2-^ structures. The dihedral angles between the C8—C9—N3 reference plane and these aromatic rings are 87.2 (2)°  for a phenyl ring and 5.4 (2)° for a pyridine ring, respectively. The dihedral angle between the phenyl ring and the pyridine ring is 92.1 (2)°. The anions and the cations are involved in C4—H4···S2, C11—H11···S2 and C9—H9···Ni weak contacts (Fig. 2, Table 2). The distances of C9···Ni and H···Ni are 3.819Å and 2.895 (2) Å, respectively, while the bond angle is 159.36 (2)° (Brookhart *et al.*,  2007).

### Experimental

The title complex was prepared by the direct reaction of 1:2:2 mol equiv. of NiCl_2_.6H_2_O, Na_2_tdas and 1-(4'-chlorobenzyl)pyridinium bromide in methanol. A brown product was given and purified through recrystallisation from the mixed solvent of methanol and water (yield 81%). The brown block single crystals suitable for X-ray analysis were obtained by slow evaporation of methanol solution of (I) at room temperature about 2 weeks.

### Refinement

All H-atoms were positioned geometrically and refined using a riding model with d(C—H) = 0.93 Å, *U*_iso_=1.2*U*_eq_ (C) for aromatic and 0.97 Å, *U*_iso_ = 1.2*U*_eq_ (C) for CH_2_ atoms.

### Crystal data

**Table:**

(C_12_H_11_ClN)_2_[Ni(C_2_N_2_S_3_)_2_]	*F*(000) = 780
*M**_r_* = 764.49	*D*_x_ = 1.611 Mg m^−^^3^
Monoclinic, *P*2_1_/*c*	Mo *K*α radiation, λ = 0.71073 Å
Hall symbol: -P 2ybc	Cell parameters from 8963 reflections
*a* = 11.3091 (10) Å	θ = 2.5–27.6°
*b* = 12.6699 (12) Å	µ = 1.21 mm^−^^1^
*c* = 12.2405 (11) Å	*T* = 296 K
β = 116.005 (1)°	Block, brown
*V* = 1576.3 (2) Å^3^	0.22 × 0.17 × 0.11 mm
*Z* = 2	

### Data collection

**Table:**

Bruker Smart AAPEX CCD diffractometer	2771 independent reflections
Radiation source: fine-focus sealed tube	2583 reflections with *I* > 2σ(*I*)
graphite	*R*_int_ = 0.023
φ and ω scans	θ_max_ = 25.0°, θ_min_ = 2.0°
Absorption correction: multi-scan (*SADABS*; Sheldrick, 2004)	*h* = −11→13
*T*_min_ = 0.788, *T*_max_ = 0.872	*k* = −15→15
11011 measured reflections	*l* = −14→14

### Refinement

**Table:**

Refinement on *F*^2^	Primary atom site location: structure-invariant direct methods
Least-squares matrix: full	Secondary atom site location: difference Fourier map
*R*[*F*^2^ > 2σ(*F*^2^)] = 0.024	Hydrogen site location: inferred from neighbouring sites
*wR*(*F*^2^) = 0.062	H-atom parameters constrained
*S* = 1.03	*w* = 1/[σ^2^(*F*_o_^2^) + (0.0288*P*)^2^ + 0.688*P*] where *P* = (*F*_o_^2^ + 2*F*_c_^2^)/3
2771 reflections	(Δ/σ)_max_ = 0.001
196 parameters	Δρ_max_ = 0.34 e Å^−^^3^
0 restraints	Δρ_min_ = −0.34 e Å^−^^3^

### Special details

**Table:**

Geometry. All e.s.d.'s (except the e.s.d. in the dihedral angle between two l.s. planes) are estimated using the full covariance matrix. The cell e.s.d.'s are taken into account individually in the estimation of e.s.d.'s in distances, angles and torsion angles; correlations between e.s.d.'s in cell parameters are only used when they are defined by crystal symmetry. An approximate (isotropic) treatment of cell e.s.d.'s is used for estimating e.s.d.'s involving l.s. planes.
Refinement. Refinement of *F*^2^ against ALL reflections. The weighted *R*-factor *wR* and goodness of fit *S* are based on *F*^2^, conventional *R*-factors *R* are based on *F*, with *F* set to zero for negative *F*^2^. The threshold expression of *F*^2^ > σ(*F*^2^) is used only for calculating *R*-factors(gt) *etc*. and is not relevant to the choice of reflections for refinement. *R*-factors based on *F*^2^ are statistically about twice as large as those based on *F*, and *R*- factors based on ALL data will be even larger.

### Fractional atomic coordinates and isotropic or equivalent isotropic displacement parameters (Å^2^)

**Table:**

	*x*	*y*	*z*	*U*_iso_*/*U*_eq_	
Ni1	1.0000	0.0000	1.0000	0.03315 (10)	
S1	0.92227 (4)	0.11624 (3)	0.85099 (4)	0.04375 (12)	
S2	0.80993 (4)	−0.08083 (4)	0.94044 (4)	0.04587 (13)	
S3	0.52625 (5)	0.07090 (6)	0.64534 (5)	0.06730 (18)	
Cl1	0.81302 (6)	0.64372 (5)	1.10601 (5)	0.06886 (17)	
N1	0.66806 (17)	0.13162 (15)	0.68113 (15)	0.0560 (4)	
N2	0.57869 (16)	−0.02144 (15)	0.75207 (15)	0.0570 (4)	
N3	0.71207 (14)	0.36476 (11)	0.59981 (13)	0.0386 (3)	
C1	0.75730 (17)	0.08146 (14)	0.77589 (16)	0.0414 (4)	
C2	0.70635 (18)	−0.00619 (13)	0.81645 (17)	0.0419 (4)	
C3	0.8199 (2)	0.57027 (15)	0.79196 (18)	0.0511 (5)	
H3	0.8155	0.6055	0.7236	0.061*	
C4	0.8134 (2)	0.62703 (16)	0.88550 (19)	0.0550 (5)	
H4	0.8048	0.7001	0.8807	0.066*	
C5	0.81978 (17)	0.57358 (15)	0.98640 (17)	0.0470 (4)	
C6	0.83425 (19)	0.46580 (16)	0.99634 (18)	0.0507 (4)	
H6	0.8390	0.4309	1.0650	0.061*	
C7	0.84157 (19)	0.41026 (15)	0.90237 (18)	0.0479 (4)	
H7	0.8525	0.3374	0.9087	0.057*	
C8	0.83301 (17)	0.46093 (15)	0.79884 (16)	0.0420 (4)	
C9	0.84261 (18)	0.39821 (16)	0.69918 (17)	0.0464 (4)	
H9A	0.8950	0.3356	0.7343	0.056*	
H9B	0.8886	0.4401	0.6636	0.056*	
C10	0.59709 (18)	0.38027 (15)	0.60462 (18)	0.0471 (4)	
H10	0.5951	0.4172	0.6695	0.057*	
C11	0.48229 (19)	0.34190 (18)	0.51417 (19)	0.0564 (5)	
H11	0.4028	0.3531	0.5178	0.068*	
C12	0.4847 (2)	0.28709 (17)	0.41842 (19)	0.0560 (5)	
H12	0.4075	0.2604	0.3572	0.067*	
C13	0.6037 (2)	0.27238 (17)	0.41461 (18)	0.0549 (5)	
H13	0.6077	0.2358	0.3504	0.066*	
C14	0.71548 (19)	0.31201 (15)	0.50599 (17)	0.0485 (4)	
H14	0.7957	0.3023	0.5033	0.058*	

### Atomic displacement parameters (Å^2^)

**Table:**

	*U*^11^	*U*^22^	*U*^33^	*U*^12^	*U*^13^	*U*^23^
Ni1	0.03566 (17)	0.02727 (16)	0.04445 (18)	0.00239 (11)	0.02487 (14)	0.00392 (11)
S1	0.0409 (2)	0.0400 (2)	0.0576 (3)	0.00339 (18)	0.0282 (2)	0.0144 (2)
S2	0.0434 (3)	0.0386 (2)	0.0568 (3)	−0.00545 (18)	0.0230 (2)	0.00919 (19)
S3	0.0467 (3)	0.0940 (5)	0.0522 (3)	0.0000 (3)	0.0134 (2)	0.0145 (3)
Cl1	0.0629 (3)	0.0786 (4)	0.0630 (3)	0.0043 (3)	0.0258 (3)	−0.0204 (3)
N1	0.0504 (9)	0.0693 (11)	0.0502 (9)	0.0087 (8)	0.0237 (8)	0.0178 (8)
N2	0.0454 (9)	0.0700 (11)	0.0526 (10)	−0.0095 (8)	0.0188 (8)	0.0016 (8)
N3	0.0401 (8)	0.0377 (7)	0.0441 (8)	0.0038 (6)	0.0240 (7)	0.0060 (6)
C1	0.0431 (10)	0.0454 (10)	0.0435 (9)	0.0059 (7)	0.0262 (8)	0.0043 (8)
C2	0.0422 (10)	0.0437 (10)	0.0450 (10)	−0.0019 (7)	0.0238 (8)	−0.0020 (7)
C3	0.0558 (11)	0.0458 (11)	0.0493 (11)	0.0039 (9)	0.0208 (9)	0.0107 (8)
C4	0.0566 (12)	0.0397 (10)	0.0609 (12)	0.0055 (9)	0.0185 (10)	0.0014 (9)
C5	0.0345 (9)	0.0545 (11)	0.0487 (11)	0.0003 (8)	0.0152 (8)	−0.0068 (8)
C6	0.0530 (11)	0.0528 (11)	0.0511 (11)	−0.0024 (9)	0.0274 (9)	0.0048 (9)
C7	0.0536 (11)	0.0390 (9)	0.0566 (11)	−0.0019 (8)	0.0292 (9)	0.0053 (8)
C8	0.0354 (9)	0.0437 (9)	0.0477 (10)	−0.0011 (7)	0.0190 (8)	0.0034 (8)
C9	0.0388 (9)	0.0537 (11)	0.0513 (11)	0.0012 (8)	0.0239 (8)	0.0035 (8)
C10	0.0431 (10)	0.0535 (11)	0.0518 (11)	0.0087 (8)	0.0274 (9)	0.0012 (9)
C11	0.0392 (10)	0.0688 (14)	0.0630 (13)	0.0102 (9)	0.0240 (9)	0.0005 (10)
C12	0.0478 (11)	0.0634 (13)	0.0510 (11)	0.0030 (9)	0.0164 (9)	0.0025 (9)
C13	0.0597 (12)	0.0629 (13)	0.0484 (11)	0.0013 (10)	0.0294 (10)	−0.0047 (9)
C14	0.0500 (11)	0.0541 (11)	0.0549 (11)	0.0028 (9)	0.0354 (9)	−0.0002 (9)

### Geometric parameters (Å, °)

**Table:**

Ni1—S2	2.1970 (5)	C4—H4	0.9300
Ni1—S2^i^	2.1970 (5)	C5—C6	1.374 (3)
Ni1—S1	2.2052 (4)	C6—C7	1.382 (3)
Ni1—S1^i^	2.2052 (4)	C6—H6	0.9300
S1—C1	1.7367 (19)	C7—C8	1.386 (3)
S2—C2	1.7351 (19)	C7—H7	0.9300
S3—N1	1.6552 (18)	C8—C9	1.499 (3)
S3—N2	1.6576 (19)	C9—H9A	0.9700
Cl1—C5	1.7428 (19)	C9—H9B	0.9700
N1—C1	1.320 (2)	C10—C11	1.374 (3)
N2—C2	1.321 (2)	C10—H10	0.9300
N3—C10	1.342 (2)	C11—C12	1.373 (3)
N3—C14	1.344 (2)	C11—H11	0.9300
N3—C9	1.505 (2)	C12—C13	1.379 (3)
C1—C2	1.436 (2)	C12—H12	0.9300
C3—C4	1.381 (3)	C13—C14	1.364 (3)
C3—C8	1.392 (3)	C13—H13	0.9300
C3—H3	0.9300	C14—H14	0.9300
C4—C5	1.382 (3)		
			
S2—Ni1—S2^i^	180.0	C5—C6—H6	120.6
S2—Ni1—S1	93.419 (17)	C7—C6—H6	120.6
S2^i^—Ni1—S1	86.581 (17)	C6—C7—C8	121.32 (18)
S2—Ni1—S1^i^	86.581 (17)	C6—C7—H7	119.3
S2^i^—Ni1—S1^i^	93.419 (17)	C8—C7—H7	119.3
S1—Ni1—S1^i^	180.0	C7—C8—C3	118.64 (17)
C1—S1—Ni1	102.38 (6)	C7—C8—C9	119.85 (17)
C2—S2—Ni1	102.90 (6)	C3—C8—C9	121.48 (16)
N1—S3—N2	98.58 (8)	C8—C9—N3	114.31 (14)
C1—N1—S3	106.72 (13)	C8—C9—H9A	108.7
C2—N2—S3	106.67 (14)	N3—C9—H9A	108.7
C10—N3—C14	120.12 (16)	C8—C9—H9B	108.7
C10—N3—C9	123.34 (15)	N3—C9—H9B	108.7
C14—N3—C9	116.44 (14)	H9A—C9—H9B	107.6
N1—C1—C2	114.09 (17)	N3—C10—C11	120.30 (18)
N1—C1—S1	124.97 (14)	N3—C10—H10	119.9
C2—C1—S1	120.93 (14)	C11—C10—H10	119.9
N2—C2—C1	113.95 (17)	C12—C11—C10	120.10 (18)
N2—C2—S2	125.75 (14)	C12—C11—H11	119.9
C1—C2—S2	120.30 (14)	C10—C11—H11	119.9
C4—C3—C8	120.74 (18)	C11—C12—C13	118.82 (19)
C4—C3—H3	119.6	C11—C12—H12	120.6
C8—C3—H3	119.6	C13—C12—H12	120.6
C3—C4—C5	118.99 (18)	C14—C13—C12	119.31 (19)
C3—C4—H4	120.5	C14—C13—H13	120.3
C5—C4—H4	120.5	C12—C13—H13	120.3
C6—C5—C4	121.56 (18)	N3—C14—C13	121.34 (17)
C6—C5—Cl1	118.66 (16)	N3—C14—H14	119.3
C4—C5—Cl1	119.76 (15)	C13—C14—H14	119.3
C5—C6—C7	118.73 (18)		

Symmetry codes: (i) −*x*+2, −*y*, −*z*+2.

### Hydrogen-bond geometry (Å, °)

**Table:**

*D*—H···*A*	*D*—H	H···*A*	*D*···*A*	*D*—H···*A*
C4—H4···S2^ii^	0.93	2.86	3.765 (2)	163
C11—H11···S2^iii^	0.93	2.80	3.715 (2)	169
C9—H9B···Ni1^iv^	0.97	2.90	3.818 (3)	159

Symmetry codes: (ii) *x*, *y*+1, *z*; (iii) −*x*+1, *y*+1/2, −*z*+3/2; (iv) −*x*+2, *y*+1/2, −*z*+3/2.
